# Rare case of refractory mixed autoimmune hemolytic anemia in a 6-year-old child: a case report

**DOI:** 10.1186/s13256-023-04154-y

**Published:** 2023-10-05

**Authors:** Mirette Hanna, Manuel Carcao

**Affiliations:** 1 Department of Laboratory Medicine and Pathobiology, University of Toronto, Toronto, ON Canada; 2 Division of Hematopathology, Department of Pediatric Laboratory Medicine, The Hospital for Sick Children, Toronto, ON Canada; 3 Research Institute, The Hospital for Sick Children, Toronto, ON Canada; 4 Division of Hematology/Oncology, Department of Pediatrics, The Hospital for Sick Children, Toronto, ON Canada

**Keywords:** Autoimmune hemolytic anemia (AIHA), Mixed autoimmune hemolytic anemia, Warm and cold autoantibodies, Case report, Pediatric

## Abstract

**Background:**

Mixed autoimmune hemolytic anemia (AIHA) shows combined clinical and laboratory characteristics of warm and cold AIHA. It is relatively uncommon in children. Consequently, knowledge about mixed AIHA prevalence, clinical presentation, treatment options, and prognosis in children is limited to very few case reports.

**Case presentation:**

We describe a six-year-old Asian girl presenting with profound anemia, blood group typing discrepancy and crossmatch incompatibility, post upper respiratory tract infection. Detection of red cell warm and cold reactive autoantibodies, led to the diagnosis of mixed AIHA. Autoantibodies with laboratory evidence of hemolysis persisted despite high dose steroid therapy. Due to the inability to wean further, the patient was subsequently commenced on mycophenolate mofetil to which she seems to be responding.

**Conclusions:**

Mixed AIHA may be notoriously difficult to diagnose and treat. Detailed clinical and laboratory work-up is essential to establish the diagnosis. To the best of our knowledge, this is the first case report of mixed AIHA following upper respiratory tract infection. Awareness of this occurrence is important, as similar to warm AIHA, mixed AIHA should be treated immediately by early initiation of steroid therapy. In addition, prompt supportive care as well as long-term clinical follow-up are required to improve outcomes of these cases.

Autoimmune hemolytic anemia (AIHA) is relatively rare in the pediatric population with the severity of the anemia being worse among children below 10 years of age [1]. AIHA is characterized by clinical and laboratory evidence of immune-mediated shortened red cell survival with direct antiglobulin test (DAT) positivity [2]. AIHA is classified as primary or secondary based on the presence of a recognized underlying cause [3]. AIHA is further subclassified into warm, cold, mixed based on the thermal characteristics of the detected autoantibody or biphasic—Donath Landsteiner type AIHA also known as paroxysmal cold hemoglobinuria (PCH) [4]. Warm reacting autoantibodies belong to IgG class and do not require complement for their hemolytic activity [4]. Instead, they result in extravascular hemolysis at a splenic level [5]. Cold reacting autoantibodies belong to IgM class, require complement for their activity and produce spontaneous red cell agglutination in vitro which reverses on warming of the sample [6]. In addition, cold reacting autoantibodies can interfere with ABO blood group typing and crossmatching with donor blood [7]. Mixed AIHA shows common characteristics of warm and cold forms [2]. PCH is characterized by binding of the biphasic IgG antibody to red blood cells (RBCs) at low temperature followed by complement binding and subsequent complement-induced intravascular hemolysis at 37 °C [6].

Warm AIHA is the most common form of AIHA diagnosed in children, accounting for approximately 70% of all cases, followed by cold AIHA, which is estimated to represent approximately 25% of all AIHA in children [8]. Mixed AIHA and PCH are the least commonly diagnosed AIHA in childhood accounting for 4% and 6% of AIHA cases occurring in children, respectively in one of the largest single institutional retrospective studies performed over a 20-year period [9]. Little is known about mixed AIHA clinical presentation, treatment options, and prognosis in children.

In this report, we describe a six-year-old girl having a clinical presentation and laboratory parameters consistent with mixed AIHA.

## Case presentation

A previously healthy six-year-old Asian girl was admitted to pediatric intensive care unit (PICU) at our institution after being referred from a community hospital for a hemoglobin of 1.6 g/dL for which she received one unit of uncrossmatched packed red blood cells (pRBCs). Approximately one week prior to presentation, the child had experienced fever, rhinorrhea, cough, and progressive fatigue. In addition, she reported having dark urine over the last three days prior to presentation. Otherwise, her past medical history was non-contributory. She had no history of blood transfusion. There was no family history of hematologic disorders. Physical examination was significant for pallor and jaundice. Chest sounds clear, with good air entry to bases. There was no palpable lymphadenopathy, organomegaly, bruises, or petechiae. Chest x-ray revealed mild peribronchial thickening in keeping with mild lower respiratory tract inflammation with no radiologic evidence of pneumonia. Abdominal ultrasound revealed mild hepatomegaly.

At presentation to our center, a complete blood count (CBC) showed: hemoglobin 2.1 g/dL (reference range (RR): 11.2–14.1 g/dL), Mean corpuscular volume (MCV) 142.9 fL (RR: 77.4–92.1 fL), hematocrit 7.0% (RR: 34.3–42.6%), White blood cells (WBC) 10.29 × 10^9^/L (RR: 4.23–9.99 × 10^9^/L) and platelet count 178 × 10^9^/L (RR: 203–431 × 10^9^/L). Reticulocyte count was 173.6 × 10^9^/L (RR: 42.4–70.2 × 10^9^/L). Peripheral blood smear examination showed severe anemia with marked red cell spherocytosis, agglutination and polychromasia. No white blood cell or platelet abnormalities were detected. The serum unconjugated bilirubin level was 35 umol/L (RR: < 7 umol/L), lactate dehydrogenase (LDH) was 2622 IU/L (RR: 470–900 IU/L), and the haptoglobin was undetectable (RR: 7–163 mg/dL).

The polyspecific and monospecific DAT showed positivity in the presence of anti-IgG and -C3d (3+), anti-IgG (3+) and anti-C3d (4+). Eluate tested against a panel of cells demonstrated panreactivity (2+). Antibody screen was positive with cell I and II (3+). Antibody identification panel by indirect antiglobulin test (IAT) showed panagglutination of uniform strength as well as a positive autocontrol (4+). Blood grouping showed 3+ agglutination with anti-A, anti-B and 6% albumin control and 4+ agglutination with anti-D, A1-cells, and B-cells in forward and reverse grouping. Prewarming of the blood sample at 37 °C for two-hours did not inhibit any of the reactions. The patient's RBCs were not serologically phenotyped due to recent transfusion at the referring hospital.

Testing for PCH by Donath Landsteiner was negative. G6PD activity was falsely increased (51.8 U/gHb, RR: 5.0–11.0 U/gHb) due to reticulocytosis interference. Rhinovirus was detected by polymerase chain reaction (PCR). Serology for cytomegalovirus (CMV), parvovirus B19, parainfluenza 1, 2, 3 and 4, respiratory syncytial virus, influenza A and B, COVID19, adenovirus was negative. Serology for mycoplasma and hepatitis C virus (HCV) was not performed.

**Fig. 1:**
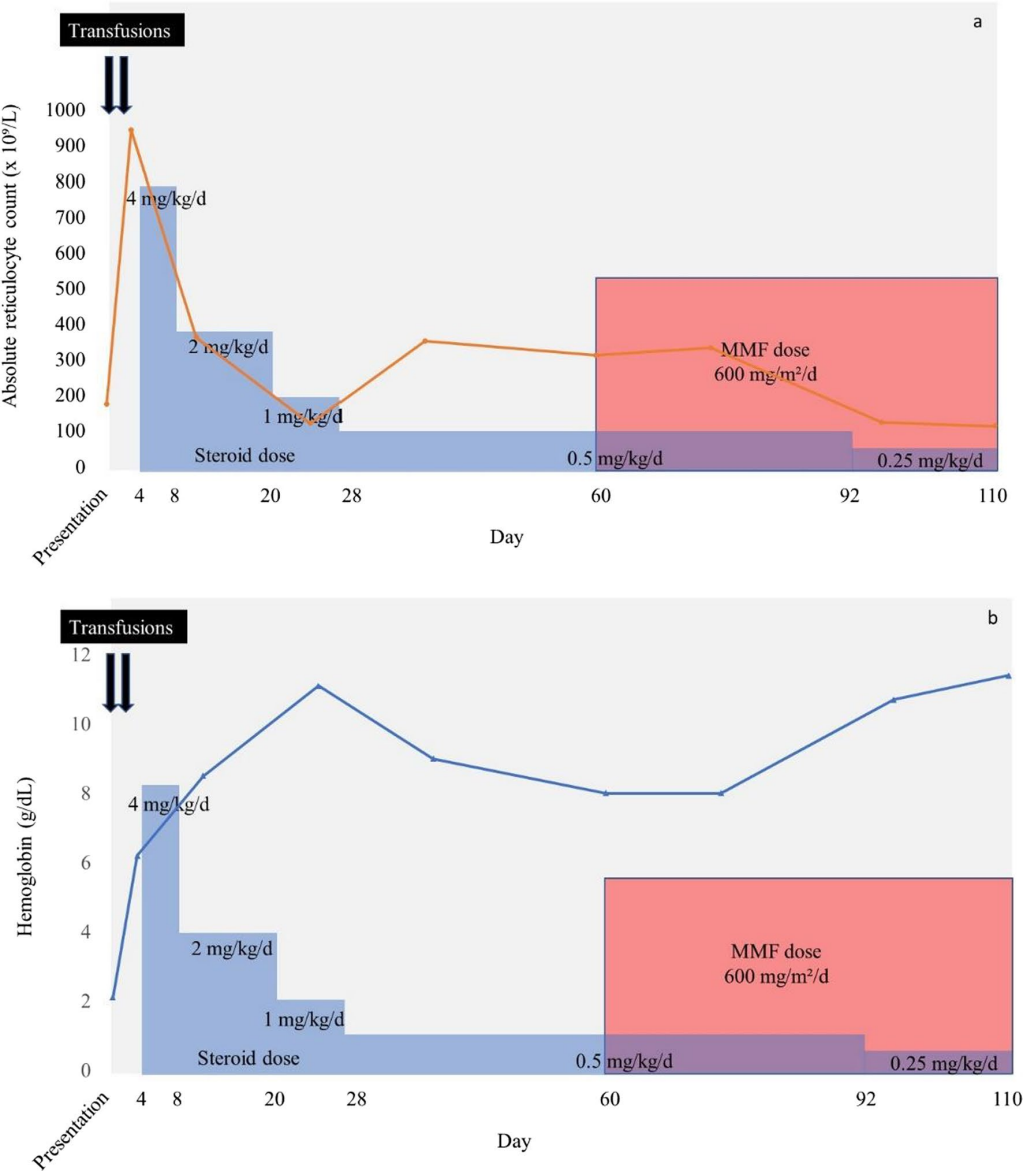
Patient treatment course and laboratory values. **a** Absolute reticulocyte count; **b** hemoglobin. Transfusions indicated as (↓)

A diagnosis of mixed AIHA was therefore made. In view of the profound anemia, the patient received group O negative crossmatch incompatible pRBCs. Transfusion was uneventful with a significant increase in hemoglobin. It was initially hoped that the hemolysis would settle but after 4 days, the reticulocyte count had risen to 942.5 × 10^9^/L and additionally the bilirubin was still rising and the hemoglobin despite transfusions was barely increasing. At this point the patient was started on oral prednisone at a dose of 4 mg/kg/day for 4 days followed by 2 mg/kg/day for 11 days. During this time, the hemolysis initially improved concurrently with a moderate decline in the reticulocyte count (Fig. 1a). This allowed for steroid dose reduction initially to 1 mg/kg/day for 7 days and then subsequently to 0.5 mg/kg/day. At the time of reducing her steroid dose to 0.5 mg/kg, her hemoglobin was 11.1 g/dL (Fig. 1b). The plan had been to reduce the steroid further, however, at this point her hemoglobin began to fall precluding further decrease in steroid dose (Fig. 1b). After 4 weeks on 0.5 mg/kg/day of steroids, her hemoglobin had fallen to 8.0 g/dL and she continued to show laboratory evidence of hemolysis. At this point, 2 months after initiation of steroid, mycophenolate mofetil (MMF) was commenced at a dose of 240 mg twice daily. Four weeks later, her hemoglobin had risen to 10.7 g/dL and her reticulocyte count had fallen to 122.8 × 10^9^/L which allowed further reduction in steroid dose to 0.25 mg/kg/day. At this point the DAT continued to be positive (2 + for the polyspecific and monospecific DAT with anti-IgG and -C3d).

## Discussion and conclusions

We report a rare case of a young girl diagnosed with mixed AIHA post upper respiratory tract infection confirmed by DAT, IAT, and peripheral blood smear examination, resulting in profound anemia, blood group typing discrepancy, and crossmatching incompatibility. Patient had uneventful transfusion with a crossmatch incompatible pRBC unit. Although she responded to high dose steroids, she could not be successfully weaned from steroids due to ongoing AIHA 2 months after initial onset.

Autoimmune hemolytic anemia (AIHA) is defined as antibody-mediated shortened RBCs survival through activation of the mononuclear phagocytic or complement system, with positive DAT, laboratory evidence of hemolysis and concurrent exclusion of alternative diagnosis [3]. The occurrence of AIHA in the pediatric population is relatively rare and presents some differences from that in adults. First, AIHA is less frequently diagnosed in children compared to adults and is estimated to occur at an incidence of approximately 0.4–0.8/100,000 child per year [1, 10]. Further, children of any age can present with AIHA [9]. Moreover, AIHA in children, much like immune thrombocytopenia (ITP) in children, often follows an infection either bacterial or viral or in some cases is seen in the setting of a systemic autoimmune disorder such as systemic lupus erythematosus (SLE). Less commonly, unlike in adults, AIHA is seen with no demonstrable underlying disease [1, 9]. Concomitant thrombocytopenia is reported in 23–37% of children with AIHA, a condition referred to as Evans Syndrome [10, 11]; some of these cases are found to have autoimmune lymphoproliferative syndrome (ALPS) [12] or to have common variable immune deficiency [13].

Laboratory evaluation of cases of AIHA can be challenging. DAT remains by far the hallmark for the diagnosis of AIHA. However, DAT is reported to be negative in up to 11% of children having warm AIHA [3]. It is worth mentioning that the strength of DAT positivity doesn’t correlate with the severity of hemolysis. Yet, DAT specificity together with the antibody thermal amplitude of reactivity helps to further subclassify the AIHA into warm (IgG only, IgG and C3 or C3 only in 20%, 67% and 13% of cases, respectively), cold (C3 with high titer, and high thermal amplitude cold agglutinins) mixed (IgG, C3 with high thermal amplitude cold agglutinins) and PCH (C3 along with positive specific Donath Landsteiner test) [4, 14, 15]. In addition, obtaining the acid eluate by detaching antibodies from RBCs should be performed to detect autoantibody specificity, although most autoantibodies typically exhibit panreactivity. Finally, adsorption studies might be worth performing to reveal masked alloantibody, however, these are time-consuming and not readily performed in all laboratories.

Children with AIHA have variable severity of anemia. Mean hemoglobin at presentation was 4.7 g/dL (SD, 1.6) in one retrospective analysis of 50 children diagnosed with AIHA [16]. Sankaran *et al.*, reported a higher hemoglobin level at diagnosis (median, 6.1 g/dL; IQR, 4.8–7.6 g/dL) [1]. They added that children aged less than 10-years had significantly lower hemoglobin levels at diagnosis compared to their counterparts (median, 5.5 vs. 7 g/dL, *P* = 0.01) [1]. Interestingly, our patient had a much lower hemoglobin of 1.6 g/dL at presentation. Mortality rate of AIHA was initially reported as high as 32% in some early series [17], mostly due to hemorrhagic complications especially in patients with concomitant thrombocytopenia, however, a relatively recent study demonstrated a much lower mortality rate of 4% [10].

Steroids are by far the mainstay of treatment of AIHA. It is worth mentioning that clinical response to steroids is not always accompanied by conversion or change in the strength of DAT [1]. In our patient, DAT remained positive for at least 2 months after initial presentation. Most children usually have an initial good response to short-term steroids, however, a relapse rate of 23.5% was reported in children ≤ 10 years after steroid tapering [1]. Second-line therapies such as intravenous immunoglobulin (IVIG) and immunosuppressive agents are reserved for refractory cases or relapsed cases after steroid weaning. There is insufficient evidence to recommend optimal duration of treatment or criteria for escalation of treatment to second-line therapy.

Transfusion of pRBCs is usually required in children with warm or mixed AIHA as the anemia tends to be severe. Transfusion should be cautiously administered due to the risk of potentiation of hemolysis and alloimmunization [6]. In addition, the presence of autoantibodies may result in ABO typing discrepancies and can further complicate crossmatching with donor blood delaying blood transfusion to the patient. Providing Rh and Kell phenotypically-matched units may reduce the risk of hemolysis due to alloantibodies masked by the autoantibody. Furthermore, if autoantibody specificity is determined, transfused RBCs should lack corresponding antigen.

Warm and cold AIHA are estimated to represent 65% and 25% of all cases of AIHA, respectively [4]. Prevalence of warm AIHA is slightly higher in the pediatric population and was reported to represent approximately 80% of childhood AIHA in one of the largest single institution series [1]. Mixed AIHA in children is rarely reported in the literature and its true incidence is unknown. It is estimated to comprise less than 5% of all AIHA cases in children [1, 9]. Owing to the very low number of reported cases, the clinical course of mixed AIHA is not revealed and patients are thought to have a chronic remitting course [18].

To date, only two patients aged less than 10-years-old diagnosed with primary mixed AIHA have been reported [19, 20]. Rai *et al.*, reported a mixed AIHA in a six-year-old female presenting with a hemoglobin of 2.8 g/dL [19]. The child had a history of pRBCs transfusion, 14 months prior to the diagnosis of AIHA. There was no evidence of underlying viral infection or immunologic disease. She received phenotypically-matched pRBCs uneventfully and was started on steroids but subsequently lost for follow-up [19]. Sheth *et al.*, reported a six-year-old girl diagnosed with mixed AIHA after presenting with fever, hepatosplenomegaly, cardiomegaly and a hemoglobin of 4.5 g/dL [20]. She responded to steroid therapy with disappearance of antibodies by 3 months of treatment [20].

Three studies reported a secondary mixed AIHA diagnosed in children less than 10 years of age [21–23]. Panzarino *et al.*, reported a mixed AIHA in a two-year-old boy occurring 8 weeks after a diagnosis of Kawasaki disease [21]. In this patient, the cold antibody showed anti-En^a^ and anti-Pr specificity [21]. Hirano *et al.*, reported a mixed AIHA secondary to SLE diagnosed in a 9-year-old girl [22]. Haller *et al.*, reported a mixed AIHA diagnosed in a one-year-old boy 2 months post isolated hepatocyte transplantation for congenital factor VII deficiency [23]. This patient had evidence of CMV and Epstein-Barr virus (EBV) infection [23]. Hemolysis resolved in the first case following treatment by steroids [21], whilst the other two patients had suboptimal responses to steroids and were switched to MMF [22] or to rituximab plus IVIG [23]. All patients received uneventful pRBCs transfusion while no episode of subsequent acute exacerbation was documented.

In summary, mixed AIHA in children, is a rare and probably underestimated disease. To date, there is limited data on the course of the mixed AIHA or the rate of exacerbation in the pediatric population. Evidence-based data is required to establish a diagnostic and treatment protocol for the AIHA, particularly mixed ones, in children.

## Data Availability

The datasets used and/or analysed during the current study are available from the corresponding author on reasonable request.

## Abbreviations

AIHA: Autoimmune hemolytic anemia
ALPS: Autoimmune lymphoproliferative syndrome
CBC: Complete blood count
CMV: Cytomegalovirus
DAT: Direct antiglobulin test
EBV: Epstein-Barr virus
HCV: Hepatitis C virus
IAT: Indirect antiglobulin test
ITP: Immune thrombocytopenia
IVIG: Intravenous immunoglobulin
LDH: Lactate dehydrogenase
MCV: Mean corpuscular volume
MMF: Mycophenolate mofetil
PCH: Paroxysmal cold hemoglobinuria
PCR: Polymerase chain reaction
PICU: Pediatric intensive care unit
pRBCs: Packed red blood cells
RBCs: Red blood cells
SLE: Systemic lupus erythematosus
WBC: White blood cells
